# From the Microbiome to the Electrome: Implications for the Microbiota–Gut–Brain Axis

**DOI:** 10.3390/ijms25116233

**Published:** 2024-06-05

**Authors:** Marwane Bourqqia-Ramzi, Jesús Mansilla-Guardiola, David Muñoz-Rodriguez, Elisa Quarta, Juan Lombardo-Hernandez, Antonio Murciano-Cespedosa, Francisco José Conejero-Meca, Álvaro Mateos González, Stefano Geuna, María Teresa Garcia-Esteban, Celia Herrera-Rincon

**Affiliations:** 1 Modeling, Data Analysis &Computational Tools for Biology Research Group, Biomathematics Unit, Department of Biodiversity, Ecology & Evolution, Faculty of Biological Sciences, Complutense University of Madrid, 28040 Madrid, Spain; marwanbo@ucm.es (M.B.-R.); jmansill@ucm.es (J.M.-G.);; 2 Department of Neurosciences “Rita Levi Montalcini”, University of Turin, 10126 Turin, Italy; 3 Unit of Microbiology, Department of Genetic, Physiology and Microbiology, Faculty of Biological Sciences, Complutense University of Madrid, 28040 Madrid, Spain; 4 Department of Molecular Biotechnology and Health Sciences, Molecular Biotechnology Center “Guido Tarone”, University of Torino, 10126 Turin, Italy; 5 Neuro-Computing and Neuro-Robotics Research Group, Neural Plasticity Research Group Instituto Investigación Sanitaria Hospital Clínico San Carlos (IdISSC), Complutense University of Madrid, 28040 Madrid, Spain; 6 University of Michigan-Shanghai Jiao Tong University Joint Institute, Shanghai Jiao Tong University, Shanghai 200240, China; 7 Department of Clinical and Biological Sciences, Cavalieri Ottolenghi Neuroscience Institute, University of Turin, Ospedale San Luigi, 10043 Turin, Italy

**Keywords:** membrane potential, microbiota–gut–brain axis, bis-(1,3-dibutylbarbituric acid) trimethine oxonol-DiBAC, growth phase, neurotransmitters, Gram-positive, Gram-negative

## Abstract

The gut microbiome plays a fundamental role in metabolism, as well as the immune and nervous systems. Microbial imbalance (dysbiosis) can contribute to subsequent physical and mental pathologies. As such, interest has been growing in the microbiota–gut–brain brain axis and the bioelectrical communication that could exist between bacterial and nervous cells. The aim of this study was to investigate the bioelectrical profile (electrome) of two bacterial species characteristic of the gut microbiome: a Proteobacteria Gram-negative bacillus *Escherichia coli* (*E. coli*), and a Firmicutes Gram-positive coccus *Enterococcus faecalis* (*E. faecalis*). We analyzed both bacterial strains to (i) validate the fluorescent probe bis-(1,3-dibutylbarbituric acid) trimethine oxonol, DiBAC4(3), as a reliable reporter of the changes in membrane potential (Vmem) for both bacteria; (ii) assess the evolution of the bioelectric profile throughout the growth of both strains; (iii) investigate the effects of two neural-type stimuli on Vmem changes: the excitatory neurotransmitter glutamate (Glu) and the inhibitory neurotransmitter γ-aminobutyric acid (GABA); (iv) examine the impact of the bioelectrical changes induced by neurotransmitters on bacterial growth, viability, and cultivability using absorbance, live/dead fluorescent probes, and viable counts, respectively. Our findings reveal distinct bioelectrical profiles characteristic of each bacterial species and growth phase. Importantly, neural-type stimuli induce Vmem changes without affecting bacterial growth, viability, or cultivability, suggesting a specific bioelectrical response in bacterial cells to neurotransmitter cues. These results contribute to understanding the bacterial response to external stimuli, with potential implications for modulating bacterial bioelectricity as a novel therapeutic target.

## 1. Introduction

The gut microbiota hosts a highly diverse community of microbes [1], with 2766 microbial species. More than 90% of the gut microbiome is represented by bacteria from the phyla Proteobacteria, Firmicutes, Actinobacteria, and Bacteroidetes [2]. Fusobacteria and Verrucomicrobia make up the remaining 10% of the gut microbiome [3]. The human gut microbiome is dramatically affected by factors such as genes, diet, and lifestyle [4]. These microbes and their genes (microbiome) play a fundamental role in metabolism [5] and the immune [6] and nervous [7] systems. Thereby, the gut microbiome has a great influence on not only physical [8] but also mental health [9]. All this has led to a growing interest in the microbiota–gut–brain (MGB) axis and the different communication pathways between the gut bacteria and neurons.

It is known that gut bacteria communicate with the central nervous system through the production of compounds such as neurotransmitters [10]. The neural-type signals generated by these bacteria are transported to the brain via afferent vagus nerve fibers, which are also linked to receptors in the esophagus, liver, and pancreas. In response to these stimuli, the brain sends signals back to enteroepithelial cells via efferent vagus nerve fibers [11] through 100 to 500 million neurons from the enteric nervous system (ENS). In addition to the widely established vagus pathway for communication along the MGB axis, bacteria can influence neurons through immune, neuronal, and metabolic pathways, and although less broadly known, through contact-mediated signaling and ion-channel-mediated electrical and chemical signaling [12,13].

The electrome, which is the sum of all the electrical activity of a living entity, from the cellular to the organismal level, is fundamental for cellular communication [14]. The movement of ions (H^+^, K^+^, Na^+^, Ca^2+^, Mg^2+^, Cl^−^, HCO^3−^), triggered by ion channels and pumps, creates a voltage gradient across the cellular membrane (Vmem). Endogenous bioelectricity is carried by these changes in the Vmem in all type of cells [15,16]. These circuits exhibit intricate dynamics among interconnected cells, constituting a layer of control with behaviors distinct from those of typical transcriptional networks. It is widely recognized across various organisms and models that bioelectricity conveys instructive information about physiological states [17,18,19,20], representing a higher physiological layer within cell populations that encompasses the interactive sum of lower levels such as genetic and metabolic pathways [21]. In eukaryotic cells, bioelectricity is involved in many processes [22], such as the regulation of neurotransmitter diffusion [23], limb regeneration [24], organ development [25], or even carcinogenic processes [26]. Far from being a unique quality for eukaryotic cells, the bacterial Vmem is dynamic, exhibiting the ability to hyperpolarize and depolarize [16]. These dynamics of the bacterial Vmem mediate signaling at both the single-cell and biofilm levels, including events such as motility [27], biofilm communication [28], cell division [29], or environmental perception [30]. Although bioelectrical signaling is becoming increasingly recognized in bacterial physiology, we still lack systematic studies characterizing the bioelectrical profiles of different bacterial strains under physiological conditions and, more importantly, in response to external cues and intercellular communication. Hence, we need to develop methods that allow analyzing the Vmem, testing in different bacterial strains, and obtaining data on bioelectrical properties under different stimuli to eventually measure the capability of endogenous and exogenous stimuli to affect bioelectrical signaling in relevant bacterial communication pathways.

We recently provided initial evidence of a functional coupling between the bioelectrical profile and growth phase in bacteria. Specifically, we investigated the changes in Vmem during the growth of *Bacillus subtilis* (*B. subtilis*) and *Limosilactobacillus reuteri* (*L. reuteri*) populations, both Gram-positive bacilli [31]. Expanding on our previous analysis, we now present, for the first time, the bioelectrical changes observed in a Proteobacterial Gram-negative bacillus, *Escherichia coli* (*E. coli*) and in a Firmicutes Gram-positive coccus, *Enterococcus faecalis* (*E. faecalis*), throughout the development of the culture, as well as in response to neurotransmitter signals. Both species can be used as probiotics modulating the gut–brain axis [32]. *E. coli*, a bacterium of the gut microbiota, is a promising probiotic platform for developing strains with the potential to regulate metabolic and multifactorial diseases [33,34,35]. The application of probiotic *E. coli* to combat gastrointestinal infections in humans is becoming increasingly important as more pathogens develop resistance to commonly used antibiotics. Additionally, some Enterococcus species, such as *E. faecalis*, are natural components of the human microbiota and are used as probiotic food additives or supplements for treating intestinal dysbiosis [36,37].

Many studies in the context of the gut–brain axis have addressed the effects of the molecules and metabolites produced by bacteria on neural cells. However, very few works have focused on the opposite aspect—specifically, the effects of neural-like signals on bacterial cells. Moreover, no studies to date have addressed the bioelectrical response of bacteria to these signals. Bioelectric signaling possesses several unique properties that render them a prime subject for investigating bacterial–neuronal communication [38,39,40,41]. This is largely due to their operation across various levels of organization [42,43,44,45,46,47], where specific bioelectric states dictate properties at the cellular, organ, and organismal levels. The bioelectrical profile offers several advantages, such as susceptibility to modification by external stimuli, without the need for genetic or biochemical manipulation. This underscores the significance of Vmem alterations and bioelectrical signaling in bacteria as promising avenues for deeper explorations within the framework of the MGB axis. Here, we established a validated methodology to explore and quantify the bioelectrical profile in bacterial populations to reveal the specific response to two widely present neurotransmitters in the MGB axis: glutamate and γ-aminobutyric acid (GABA). Crucially, we found that neural-type stimuli induce Vmem changes without affecting bacterial growth, viability, or cultivability, suggesting a specific bioelectrical response of bacterial cells to neurotransmitter cues.

## 2. Results

### 2.1. The Voltage-Sensitive Dye DiBAC Is a Reliable Marker for Detecting Membrane Potential (Vmem) Changes in E. coli and E. faecalis Cells

**Figure 1:**
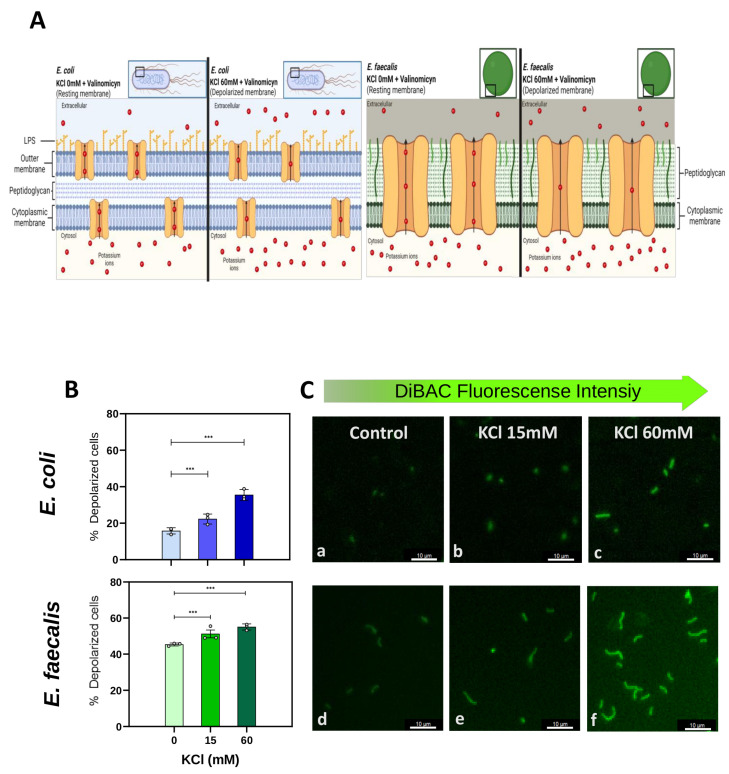
DIBAC validation as a tool for measuring Vmem in *E. coli* and *E. faecalis*. (**A**) Conceptual schematic of DiBAC validation assay. Increasing concentrations of KCl (in presence of valinomycin, Val) were added in the extracellular medium to induce depolarization (due to a lower efflux of K^+^ ions). Created with BioRender.com (Toronto, ON Canada) (**B**) Quantification of DiBAC fluorescence using an ImageJ macro (National Institutes of Health, Bethesda, MD, USA) . Comparation of percentage of depolarized cells in presence of different KCl concentrations (control or 0 mM, 15 mM, or 60 mM), applying the generalized estimating equations (GEEs) statistical method. A significant increase in the percentage of depolarized cells was observed as the KCl increased in the extracellular medium. *** *p*-values (*p* < 0.01). For each experimental condition, values from three biological replicates (dots) with at least three technical replicates each are plotted. (**C**) Epifluorescence microscopy images. High-magnification images show DiBAC-expressing cells (in green) of *E. coli* (a–c) and *E. faecalis* (d–f)) for control (a,d), KCl 15 mM (b,e), and KCl 60 mM (c,f). Scale bar = 10 µM.

First, we validated the fluorescent voltage-sensitive dye DiBAC as a tool to determine and quantify the changes in the Vmem in the *E. coli* and *E. faecalis* bacteria (Figure 1). We exposed cells to known and increasing concentrations of potassium chloride (KCl; 0 mM or control–15 mM–60 mM) and the K^+^-ionophore/antibiotic valinomycin (Val; 5 µM). Then, we stained bacteria with DiBAC dye, which has a negative charge and is stored within depolarized cells, to quantify the fluorescence intensity per each population (imaging at single-cell resolution). We set thresholds for depolarization at the average DiBAC fluorescence intensity in the control condition and compared the percentage of cells above the depolarization threshold in each experimental condition.

We found that both *E. coli* and *E. faecalis* behaved similarly in response to increasing extracellular KCl. In *E. coli* (Figure 1B,C, top row), the percentage of depolarized cells (DiBAC-positive cells above the threshold) increased from 15.79 ± 1.13% in the control to 22.31 ± 1.60% in KCl15 and further to 35.52 ± 3.28% in KCl60. Statistical analysis showed significant differences in the % of depolarized cells among the three groups (multilevel logistic regression model; MLoRM: *p*-values < 0.001 between control and KCl15, and control and KCl60; for detailed statistics, see Supplementary Table S1), demonstrating that experimentally induced depolarization in *E. coli* is revealed by an increase in DiBAC fluorescence.

Similarly, in *E. faecalis* (Figure 1B,C, bottom row), the percentage of depolarization increased from 45.35 ± 1.93% in the control to 51.22 ± 2.86% in KCl15 and further to 55.07 ± 1.67% in KCl60. Statistical analysis revealed statistically significant differences in the % of depolarized cells among the three groups (MLoRM: *p*-values < 0.001 between control and KCl15, and control and KCl60; for detailed statistics on *p*-values, regression coefficients, and confidence intervals, see Supplementary Table S1), demonstrating that DiBAC is also a reliable marker for depolarization in *E. faecalis*.

In addition, we built a regression model using the quantitative properties of KCl to enhance forecasts for the percentage of depolarized cells, *p*, for any concentration of KCl expressed in mM [KCl],

$$p=\frac{1}{1+e^{-\left(\alpha_{0}+\alpha_{1}[KCl],\right)}}$$

where $\alpha_{0}\;$and $\alpha_{1}$ are the specific values for each bacterium:

*E. coli*: $\alpha_{0}=-1.614883$, $\alpha_{1}=0.0158313$; *E. faecalis*: $\alpha_{0}=-0.0078862$, $\alpha_{1}=0.001233$.

Taken together, we conclude that the fluorescence intensity of the voltage indicator DiBAC is clearly higher when cells are more depolarized (induced by controlled conditions of extracellular K^+^) in both *E. coli* and *E. faecalis*, validating and quantifying this morphological approach for readings of Vmem changes in bacterial cells.

### 2.2. Changes in Vmem during Growth Phases Are Unique for Each Bacterial Strain

Having validated the voltage-sensitive dye DiBAC as a reporter for Vmem changes, we decided to study and quantify the bioelectrical characteristics of both *E. coli* and *E. faecalis* during the different stages of growth. To do this, we conducted DiBAC assays and imaging analysis of samples collected at different time intervals from the inoculation (t = 1 h, t = 3 h, and t = 5 h), representing the early, middle, and late exponential phases, respectively. Additionally, we calculated the cell growth rate (rt) at each specific time point to relate depolarization and growth dynamics (refer to the Supplementary Information for a detailed explanation and calculations). The threshold for depolarization was set as the mean value of DiBAC fluorescence intensity at the first measured time (t = 1 h).

**Figure 2:**
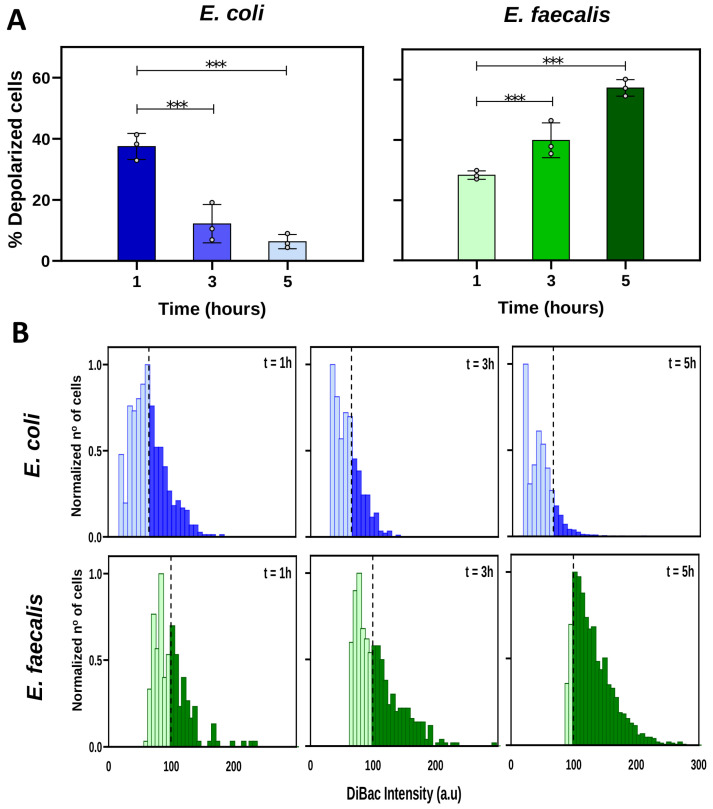
Bioelectrical profile throughout bacterial growth. Axenic cultures of *E. coli* or *E. faecalis* were prepared in fresh TSB medium and incubated for 5 h. At t = 1 h, t = 3 h, and t = 5 h, bacterial cells were sampled and stained with DiBAC to reveal the membrane potential (Vmem) by epifluorescence microscopy. Generalized estimating equations (GEEs) were applied as the statistical method. (**A**) Percentage of depolarized bacteria. Values from three biological replicates (dots) with three technical replicates for each condition are represented per time. The percentage of depolarized cells decreased significantly with growth time in *E. coli*, and, on the contrary, it increased in *E. faecalis* (*** *p* < 0.001). The percentage of depolarized cells in *E. coli* varied from 37.48 ± 2.45% at t = 1 h to 12.18 ± 3.61% at t = 3 h and further reduced to 6.33 ± 1.34% at t = 5 h. Simultaneously, values for growth rate of *E. coli* population at each time point were rt = 1.34, 0.65, and 0.07 at 1, 3, and 5 h, respectively. The percentage of depolarized cells in *E. faecalis* varied from 28.31 ± 0.81% at t = 1 h to 43.22 ± 6.62% at t= 3 h and further to 57.27 ± 1.58% at t = 5. Values for growth rate of *E. faecalis* culture at each time point were rt= 1.05, 0.81, and 0.28 at 1, 3, and 5 h, respectively (for detailed statistics, see Supplementary Table S2). (**B**) Frequency distribution histograms of DIBAC expression in both bacteria. Data are plotted as the total number of cells, normalized to the number of those exhibiting the most frequent intensity value at each of the three time points. Depolarization threshold (average DiBAC fluorescence intensity at t = 1 h) was set at 65.32 and 99.82 arbitrary units (a.u) for *E. coli* and *E. faecalis*, respectively (dashed line).

In *E. coli*, the proportion of depolarized cells (above the depolarization threshold) decreased as the growth period advanced (Figure 2A left). Conversely, in *E. faecalis* we detected a clear increase in the proportion of depolarized cells (above the depolarization threshold) as the growth period advanced (Figure 2A right).

Furthermore, to explore the bioelectrical profile within each population, we analyzed the frequency distribution of DiBAC fluorescence intensities over time, representing the data normalized to the number of cells whose intensity value was the most frequent at each time (Figure 2B). Depolarization thresholds were set at 65.32 and 99.82 arbitrary units (a.u., dashed line) for *E. coli* and *E. faecalis*, respectively. This analysis, based on fluorescence microscopy images, revealed a gradual and specific shift in the distribution curve for each bacterial strain over time. The curve for *E. coli* shifted leftward, indicating a decreasing number of bacterial cells above the depolarization threshold, while the curve for *E. faecalis* shifted rightward, indicating an increasing number of cells above the depolarization threshold over time.

Taken together, these results indicate that *E. coli* and *E. faecalis* exhibit distinct DiBAC-measured bioelectrical profiles over the course of the culture period. Specifically, while depolarization decreases with time in *E. coli*, it increases with time in *E. faecalis*.

### 2.3. Glutamate and GABA Exposure Induces Vmem Changes in Bacteria

After demonstrating that both *E. coli* and *E. faecalis* display dynamic depolarization profiles during the different growth stages (changes in physiological states), we next investigated whether an external stimulus with neural properties could also influence the Vmem of these bacterial cells. Bacteria were incubated for 4.5 h (until reaching the late-exponential phase) in the presence of glutamate (Glu) and GABA. Then, their bioelectrical profiles were measured using DiBAC staining and compared to the control group (without drug treatment; threshold for depolarization was set at the mean value for DiBAC intensity in the control group; refer to Supplementary Figure S1E for a schematic model).

**Figure 3:**
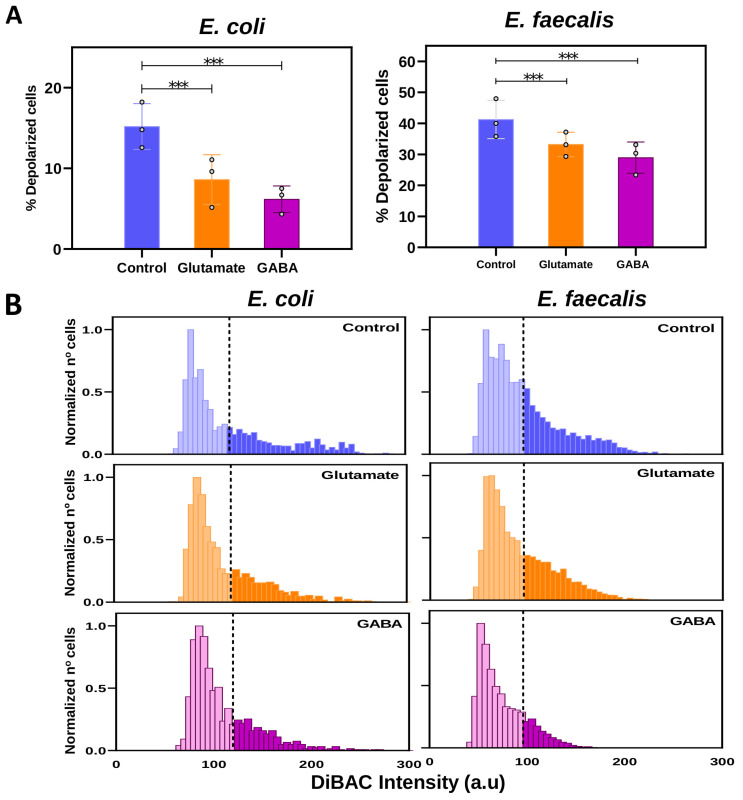
The effect of neurotransmitters on bacteria bioelectricity. Axenic cultures of *E. coli* or *E. faecalis* were prepared in fresh TSB medium and incubated for 4.5 h without (control) or with neurotransmitters (75 µM glutamate or 0.01 µM GABA). Subsequently, the bioelectrical activity of the bacteria in each group was measured using DiBAC as a membrane potential (Vmem) reporter. Analysis by epifluorescence microscopy was made, and generalized estimating equations (GEEs) were applied as the statistical method. (**A**) Percentage of depolarized bacteria. Values from three biological replicates (dots) with three technical replicates for each condition are represented per time. Both neurotransmitters induced a significant decrease in the percentage of depolarized *E. coli* or *E. faecalis* compared to the control (*** *p* < 0.001). (**B**) Frequency distribution histograms of DIBAC expression in both bacteria. Data are plotted as the total number of cells, normalized to the number of those exhibiting the most frequent intensity value in each treatment (control, blue; glutamate, orange; GABA, pink). Depolarization threshold (average DiBAC fluorescence intensity value of control cells) was setting at 119.75 and 96 arbitrary units (a.u) for *E. coli* and *E. faecalis*, respectively (dashed line).

In *E. coli*, the proportion of depolarized cells (above the depolarization threshold) decreased when cells were treated with neurotransmitter drugs (Figure 3A left). Specifically, the percentage of depolarized cells in *E. coli* lowered from 14.43 ± 2.29% in the control group to 8.61 ± 1.71% in the Glu-treated and 6.17 ± 0.95% in the GABA-treated groups. Statistical analysis revealed that these differences were statistically significant both between the control and Glu, and between the control and GABA groups (MLoRM: *p* < 0.001 for both cases; for detailed statistics on *p*-values, regression coefficients, and confidence intervals, see Supplementary Table S3).

Likewise, in *E. faecalis*, the percentage of depolarization decreased when bacteria were exposed to either Glu or GABA (Figure 3A right). The values for % depolarized cells (above the threshold) ranged from 41.24 ± 3.46% in the control to 33.22 ± 3.90% in the Glu-treated and 28.94 ± 3.35% in GABA-treated cultures. Statistical analysis showed significant differences among treatments (MLoRM: *p* <0.001 for both control vs. Glu, and control vs. GABA; for detailed statistics on *p*-values, regression coefficients, and confidence intervals, see Supplementary Table S3).

To further analyze the depolarization profile of the differently treated cultures, we examined their cell distribution based on fluorescence intensities (Figure 3B). Both bacteria behaved similarly: the curve of the control exhibited the most widespread distribution, with discernible differences in number and shape above the depolarization threshold at 119.75 and 96 a.u. (dashed line) for *E. coli* and *E. faecalis*, respectively, compared to Glu- and GABA-treated cultures. In both studied bacteria, the distribution curve, representing the depolarized cell population in Glu and GABA treatments, appeared to shift leftward compared to that of the control population, indicating a decrease in DiBAC fluorescence intensity.

We conclude from these results that exposure to Glu and GABA alters the bioelectrical properties of both *E. coli* and *E. faecalis* populations. Notably, both populations show a similar response, characterized by a decrease in the proportion of depolarized bacteria when cultivated in the presence of these neurotransmitters (both glutamate and GABA), compared to the control or untreated group.

Next, to ascertain whether Glu and GABA specifically altered the Vmem, or if this effect was indirect (potentially mediated through neurotransmitter impacts on the cell cycle or growth), we proceeded to selectively assess various aspects of bacterial biological activity under the influence of these neurotransmitters. Both bacteria were incubated in the presence of Glu or GABA for 6 h, during which we analyzed the growth dynamics, cultivability, and bacterial viability.

**Figure 4:**
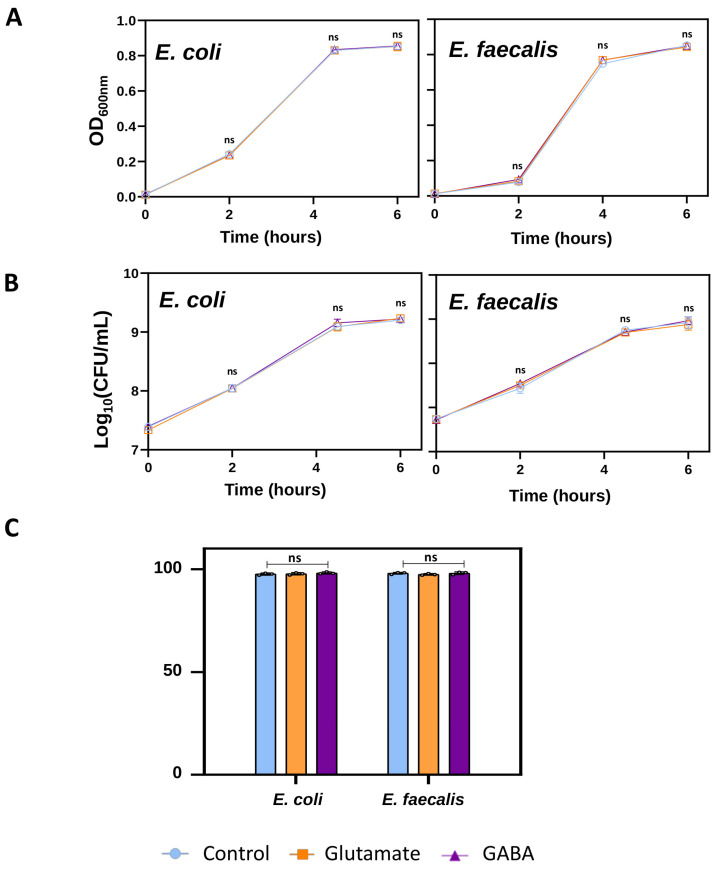
The effect of neurotransmitters on bacterial growth, cultivability, and viability. (**A**) Both bacterial strains were incubated with glutamate and GABA for 6 h, during which their growth dynamics were analyzed by measuring optical density at 600 nm (OD600). (**B**) The cultivability was assessed by viable count (CFU/mL). (**C**) Bacterial viability was determined using a LIVE/DEAD™ BacLight™ Bacterial Viability Kit to count live and dead cells. Data from three biological replicates (dots) with three technical replicates for each condition are presented for each time point. Neither neurotransmitter caused a significant change in OD600, CFU/mL, or the percentage of live/dead cells (*p* > 0.05).

We found similar results for *E. coli* and *E. faecalis* in the three measurements. For both *E. coli* and *E. faecalis*, growth curves (built from the optical density measurements taken throughout the 6 h period, at t = 2 h, t = 4.5 h, and t = 6 h) displayed similar patterns among the three experimental groups (control or untreated vs. Glu-treated, and vs. GABA-treated). The statistical analysis revealed no significant differences in the OD600 values among groups at any given time (multilevel linear regression model, MLiRM: *p* > 0.05 for all cases; Figure 4A; for detailed statistics on *p*-values, regression coefficients, and confidence intervals, see Supplementary Tables S4 and S5).

Likewise, the cultivability or viable counts (CFU/mL) for *E. coli* and *E. faecalis* showed no differences among control or untreated, Glu-treated, and GABA-treated bacterial cells, with similar values at t = 2 h, t = 4.5 h, and t = 6 h (MLiRM: *p* > 0.05 for all cases; Figure 4B; for detailed statistics on *p*-values, regression coefficients, and confidence intervals, see Supplementary Tables S6 and S7).

Then, we measured the percentage of live cells by determining the numbers of viable and nonviable cells using a LIVE/DEAD™ BacLight™ Bacterial Viability Kit at t = 4.5 h, which corresponds to the time when we evaluated the DiBAC images. Strikingly, we detected no changes in viability when cells were grown in Glu or GABA for 4.5 h (MLiRM: *p* > 0.05 for all cases; Figure 4C; for detailed statistics on *p* -values, regression coefficients, and confidence intervals, see Supplementary Table S8).

Considering all these data, we claim that the presence of the neurotransmitters Glu or GABA has no effects on bacterial physiological events such as growth, cultivability, and viability. Conversely, neurotransmitter exposure has clear effects on the bioelectrical of both *E. coli* and *E. faecalis*. We conclude that Glu and GABA exposure induces specific Vmem changes in bacteria without altering other biological properties.

## 3. Discussion

In this study, we demonstrated that the bioelectrical properties of two different bacteria, *E. coli* and *E. faecalis*, exhibit specific profiles in response to physiological events, such as growth dynamics, and exposure to external stimuli, such as neurotransmitter drugs. Utilizing a membrane potential (Vmem) reporter, DiBAC, combined with microscopy and imaging analysis, we revealed that the proliferative cells in *E. coli* and *E. faecalis* expressed bioelectrical profiles in apparent opposite directions. While the *E. coli* population experienced a decrease in depolarization as culture time progressed, the *E. faecalis* population showed an increase in the percentage of depolarized cells over time. Both bacterial strains responded similarly to the presence of the neurotransmitters glutamate (Glu) and GABA, resulting in a reduction in the overall depolarization of the culture. These Vmem changes in response to Glu and GABA were not attributed to the indirect effects of these drugs on bacterial physiology, as they did not influence cultivability or viability.

The bioelectrical profile in bacteria is increasingly recognized as a remarkable property for these cells [29,48,49,50,51], correlated with significant physiological events such as motility [27], biofilm communication [28], cell division [29], and environmental perception [30]. Electrical signaling in bacteria has been evaluated using different approaches. Kralj et al. were pioneers in probing the electrical spiking in *E. coli* using a voltage-sensitive fluorescent protein based on green-absorbing proteorhodopsinin [48]. Some years later, Bruni et al. [49] developed a genetically encoded calcium reporter to show that voltage-dependent calcium influx mediates the response to mechanical cues in *E. coli* (similar to sensory neurons in vertebrates). A number of voltage-sensitive fluorescent Nerst-type probes have been used to follow depolarization/hyperpolarization in bacteria, including 3,3′-dipropylthiadicarbocyanine iodide (DiSC3(5)), 3,3′-diethyloxacarbocyanine iodide (DiOC2(3)), thioflavin T (ThT), and DiBAC [50]. In this work, we validated and characterized the use of DiBAC as a reporter for Vmem changes in both a Gram-negative bacillus (*E. coli*) and a Gram-positive coccus (*E. faecalis*). Both *E. coli* and *E. faecalis* are key representatives of the gut microbiota and can be utilized as probiotics to influence the gut–brain axis [32]. *E. coli*, in particular, holds promise as a probiotic platform for developing strains that may help regulate metabolic and multifactorial diseases. Several studies have reported the in vitro inhibitory effects of certain *E. coli* isolates on the growth of Shiga-toxin-producing STEC strains (EHEC) [33,34]. Additionally, *E. coli* forms the basis of several commercially available probiotic products aimed at combating gastrointestinal infections, especially as antibiotic resistance in pathogens becomes more prevalent [35]. Similarly, Enterococcus species like *E. faecalis* are natural components of the human microbiota and are used as probiotic food additives or supplements. They are particularly beneficial for treating intestinal dysbiosis [36] and acute diarrhea [37].

First, we validated DiBAC in both *E. coli* and *E. faecalis* by evaluating its ability to detect Vmem changes when cells are grown in a controlled depolarizing medium (Figure 1). Overall, these techniques work well in many Gram-positive bacteria [31]. However, fluorescent probes such as Vmem reporters generate low signal-to-noise ratios in Gram-negative bacteria [52] due to dye exclusion by the outer membrane [53]. We verified, as previously reported [54], that in *E. coli*, pretreatment with EDTA was necessary for visualizing DiBAC. EDTA chelates divalent cations, stabilizing the lipopolysaccharide molecules of the outer membrane, allowing dye access to the inner membrane [55]. Bacterial cells employ active potassium (K^+^) transport mechanisms to concentrate intracellular K^+^ ([K^+^]in) at approximately 300 mM [56], a level nearly 40 times higher than the extracellular K^+^ concentration ([K^+^]out). The resting Vmem of these cells typically resides around −90 mV. Upon adding extracellular K^+^ (and increasing the K^+^ membrane permeability using the ionophore valinomycin), the electrical potential difference across the membrane decreases, and, thus, cells are depolarized (Figure 1A). DiBAC is an anionic lipophilic fluorescent probe that only enters in the cell when its membrane depolarizes, becoming positively charged. As the inner leaflet becomes more positive, more DiBAC enters, and the DiBAC fluorescent signal becomes more intense [57] due to the binding to positively charged intracellular proteins or to the hydrophobic regions [58].

Once we verified DiBAC as a reporter for Vmem changes in these bacterial strains, we characterized the dynamics of the Vmem in *E. coli* and *E. faecalis* throughout their growth curve (Figure 2). Our results show an increase in the percentage of depolarized cells in *E. faecalis* with population growth. Gram-positive bacilli demonstrated similar behavior to that in previous studies [31]. On the contrary, we observed a decrease in the percentage of depolarized cells in *E. coli* throughout its growth. Some authors have demonstrated that changes in voltage alter the elongation and division of *E. coli* [54]. Previous studies of bacterial cell division identified the membrane potential as a key parameter in controlling the localization of the proteins necessary for cell division [29]. Similarly, Vmem has been linked to cell wall synthesis, mediated by turgor pressure and membrane tension [59,60]. Although more studies are necessary to understand the influence of Vmem on bacterial growth and to verify and understand possible differences between Gram-positive and Gram-negative bacteria, our results suggest that bioelectrical profiles displayed at the different growth phases are strain-specific. Interestingly, we observed significant differences between *E. coli* and *E. faecalis* from the onset of population growth, with a notably higher percentage of depolarized cells in *E. coli* (which decreased as time progressed).

The last part of our study analyzed whether external stimuli can modify the bioelectrical properties of bacteria without altering other physiological events. Bacteria produce and/or modulate neurotransmitters, which affect neurons [10,61]. However, the effect of these neural-type signals on bacteria is poorly understood. Among these signals, neurotransmitters stand out because they participate in the transmission of information through the nervous system in addition to influencing other processes such as morphogenesis [62], embryogenesis, and regeneration [63], as well as the immune system [38]. We studied the effect of the two main neurotransmitters of the CNS widely present in the MGB axis: Glu and GABA. Glu is the principal excitatory neurotransmitter in the brain and plays a key role in memory storage [64]. In fact, excessive Glu accelerates the progression of Alzheimer’s disease [65]. In addition, Glu mediates electrical communication in bacterial communities [66], inducing K^+^ efflux, which depolarizes neighbor cells. A similar effect was observed in plants [67]. GABA is an inhibitory neurotransmitter that can be produced by gut bacteria [68] or even utilized by bacteria such as *E. coli* as a carbon and nitrogen source [69]. Both neurotransmitters have antagonistic effects in the nervous system of mammals: Glu has typically a depolarizing effect, while GABA acts as a hyperpolarizing signal. In our study, however, both glutamate and GABA significantly decreased the percentage of depolarized bacteria (Figure 3). This is not the first time that antagonistic drugs have induced similar effects outside of the nervous system of mammals. Sullivan et al. obtained the same craniofacial and patterning defects in Xenopus in the presence of agonist and antagonist drugs [63]. More recently, Muñoz et al. also observed a decrease in depolarization for Gram-positive bacilli (*L. reuteri* and *B. subtilis*) in the presence Glu or GABA [31]. In addition, we observed a slightly higher depolarization threshold in *E. coli* than in *E. faecalis* (Figure 3B). Yet, the reduction in the proportion of depolarized cells induced by neurotransmitters was greater with GABA, especially in *E. faecalis*. In addition, changes in the endogenous bioelectricity of the bacteria induced by both neurotransmitters were not accompanied by significant modifications in the growth or the number of viable bacteria or the number of viable culturable bacteria (Figure 4). Future studies should address whether the neurotransmitter-induced decrease in the depolarization described in this article is activating or inhibiting bacteria.

This is the first observation of the influence of nervous signals on the bioelectrical profile of Gram-negative bacilli and Gram-positive cocci, representative of the gut microbiota. Additionally, this study is the first to demonstrate that these changes do not result in variations in the growth, viability, or cultivability of the bacteria. The significance of the results obtained justifies the need for further studies that include a broader range of bacterial species and consider the potential impact of varying experimental conditions. Additionally, it is crucial to investigate the regulatory mechanisms underlying these processes. In this context, our next objective is to elucidate the channels, effectors, and downstream pathways that mediate this relay. The distinct Vmem response dynamics and its quantification by DiBAC offer a valuable proof of concept for bioelectrical signaling, which can be applied to analyze the response of bacterial cells to neural stimuli, particularly relevant in the context of the microbiota–gut–brain axis.

## 4. Materials and Methods

### 4.1. Bacterial Strains and Culture Conditions

All the bacterial strains used in this study were obtained from the ATCC (American Type Culture Collection; LGC Standards, Barcelona, Spain). *E. coli* NCTC 9001 was used as a Gram-negative bacterium, and *E. faecalis* ATCC 19433 was used as a Gram-positive bacterium. To prepare pure culture, frozen bacteria stored at −80 °C were precultured in the corresponding medium, tryptic soy broth (TSB), for 12 h at 37 °C. Then, 50 µL of each bacterium in glycerol solution was added to 5 mL of TSB. After overnight growth, a dilution of 1:500 was prepared by adding 10 μL of the preculture to 5 mL of fresh medium (subculture). Using 10 μL disposable loops, Petri dishes with the corresponding agar trypticase soy agar (TSA) were also prepared to obtain isolated colonies. Both broth and dish preparations were incubated for 24 h at 37 °C. Then, each culture was centrifuged at 6000× *g* for 20 min and prepared for subsequent experimental conditions. The growth dynamics of both bacteria were previously characterized using a spectrophotometer (Thermo electron corporation, Helios Epsilon, CAT: 9423UVE1000E; Thermo Fisher Scientific, Madrid, Spain), taking measurements of optical density at 600 nm (OD600) and cultivability by viable count (CFU/mL) every 30 min or 1 h. Three independent biological replicates (with three technical replicates each) were used to build the growth curve (Supplementary Figure S1A–D). the late exponential phase (at 4.5 h) was chosen as the reference stage for most of the experiments (when others or different time points were used, a statement is clearly included).

### 4.2. Validation of (DiBAC4(3)), a Voltage-Sensitive Fluorescent Dye, in Bacterial Cultures

To ascertain the effectiveness of the fluorescent dye bis-(1,3-dibutylbarbituric acid)-trimethine oxonol (DiBAC4(3)) (DiBAC; Fisher Scientific ref. B438; Madrid, Spain) as an indicator of membrane voltage changes, bacteria were exposed to a depolarizing medium, achieved by elevating extracellular potassium ion concentrations ([K^+^]out) with KCl (Figure 1). To enhance the permeability of the cell membrane to K^+^ ions, 5 µM valinomycin (Val; Fisher Scientific ref. V1644; Madrid, Spain) was employed as an ionophore or K^+^ carrier. Specifically, KCl was added incrementally until [K^+^]out levels of 15 mM and 60 mM were reached, corresponding to Vmem values of approximately −75 mV and −40 mV, respectively [70]. The resulting DiBAC profiles were then compared to those of the bacteria under resting conditions (Vmem approaches the equilibrium potential for potassium (Veq K^+^) around −110 mV; see Supplementary Information for a description of the Nernst equation).

Both bacterial species underwent overnight cultivation at 37 °C under aerobic conditions and were subsequently subcultured onto fresh culture medium for a period of 12 h. Following this, the OD600 was measured and adjusted to OD600 = 0.3. The bacteria were then separated from the culture medium through centrifugation (2000× *g*, 10 min, room temperature (RT)). Then, *E. faecalis* was resuspended in 1x phosphate-buffered saline (PBS) while maintaining the bacterial concentration. Since *E. coli* is a Gram-negative bacterium, a pretreatment was required to facilitate the passage of DiBAC through its membrane [48]. To this end, *E. coli* cells were incubated in 1 mM EDTA (Tris-Borate-EDTA; Fisher Scientific, ref: BP1333-1; Madrid, Spain) for 1 min at RT, followed by centrifugation (2000× *g*, 10 min, RT), and resuspension in 0.1 mM EDTA. Subsequently, bacterial cells were plated in a standard 48-well plastic plate with 5 µM Val diluted in TSB. Bacterial wells were randomly divided into the three experimental groups: control or no KCl added (control), 15 mM of added KCl (KCl15), and 60 mM of added KCl (KCl60). Then, cells were stained with the optimized conditions of DiBAC. When using a voltage-sensitive dye on new bacterial species, the optimal dye concentration and incubation time should first be determined, as extensively shown in [31,70], as different species do not respond equally to the same dye conditions. The selection of DiBAC concentration, incubation time, and temperature was determined through multiple optimization experiments with the respective bacteria. For *E. coli*, DiBAC was employed at a concentration of 50 μM, while, for *E. faecalis*, a concentration of 10 μM was utilized. The incubation period for both was 10 min at RT in the dark. After incubation, cells were prepared for microscopy and imaging.

### 4.3. DiBAC4(3) Assay to Characterize Bioelectrical Profile during Growth 

Both bacteria were grown overnight with O_2_ at 37 °C in TSB and subcultured in fresh medium for 12 h. The OD600 of the culture was then adjusted to 0.01 in fresh TSB and incubated at 37 °C. After 1 h (early exponential phase), 3 h (mid exponential phase), and 5 h (late exponential phase) of incubation, a 1 mL sample was taken to perform the DiBAC analysis (Figure 2). Cells were centrifuged (2000× *g*, 10 min, RT), resuspended in 1× PBS (*E. coli* was pretreated in EDTA), diluted to an OD600 ~0.3, and placed in a 48-well plate with DiBAC, as set out in the previous section. Three biological replicates with at least three technical replicates each, were evaluated at each timepoint. For each technical replicate and timepoint, several photographs, both phase contrast images and fluorescence filters, were taken to quantify the percentage of depolarization at the different states of the growth dynamics.

### 4.4. Effect of Neurotransmitters on E. coli and E. faecalis: Bioelectrical Profile, Growth, Cultivability, and Viability

The preparation of both *E. coli* and *E. faecalis* cultures was performed as described above. OD600 was measured and adjusted to 0.01 in fresh TSB media. For glutamate (Glu) and GABA assays, cell suspensions were supplemented with 75 µM of glutamate (Tocris-Biotechne, Bio-Techne R&D Systems, S.LU, ref. 0218; Madrid, Spain) and 0.01 µM of GABA (Tocris-Biotechne, Bio-Techne R&D Systems, S.LU, ref.0344; Madrid, Spain), respectively, and incubated at 37 °C under same conditions (Supplementary Figure S1E). Drugs were previously dissolved in HEPES 1M (Thermo Fisher Scientific, ref. 15630106, Madrid, Spain). Drug concentrations were determined using ranges supported by the supplier and through dose screening and were set at levels that did not result in observable toxic effects. Both bacteria were incubated in the presence of Glu and GABA for 6 h, during which growth dynamics, cultivability, and the bacterial viability were analyzed by measuring optical density (OD600), viable count (CFU/mL), and determining the number of alive and dead cells using a LIVE/DEADTM BacLightTM Bacterial Viability Kit (Figure 4). OD600 and cultivability were evaluated over the course of the experiment, with measurements at 2 h, 4.5 h, and 6 h (Supplementary Figure S1E). For cultivability, the sample was taken at 10^−3^ dilution, diluted to 10^−6^ in 1× PBS, and seeded by drop plate method in Petri dishes with TSA. Petri dishes were left overnight at 37 °C until colonies could be counted. At least 10 drops were counted at the selected dilution. After 4.5 h of drug incubation, cells were centrifuged (2000× *g*, 10 min, RT), resuspended in 1× PBS diluting the sample to an OD600 ~0.3 (*E. coli* was pre-treated in EDTA), and placed in a 48-well plate with DiBaC, as set out in the previous section, to evaluate the bioelectrical properties of the culture under the action of the neurotransmitters. After the incubation, epifluorescence microscopy analysis was performed (Figure 3). Untreated cells were established as a control group. We performed the live/dead test at 4.5 h (corresponding to the timepoint when the bioelectrical profile was detected), following protocols described by the manufacturer. The images were then taken using a Leia DMi8 microscope. To detect live cells, we used excitation at a wavelength of 480–490 nm. To detect dead cells, we used excitation at 515–560 nm. Percentage of alive cells for each group, control, Glu-treated and GABA-treated, was estimated to plot as 100—ratio red/green.

### 4.5. Imaging and Image Analysis

After DiBAC staining (validation, growth dynamics, and neurotransmitter assays), cells were prepared for microscopic analysis. To this end, 5 µL of the DiBAC-stained cell solution was applied onto a microscope slide and covered with a 19 mm diameter microscopy coverslip. An inverted Leica DMi8 microscope (Leica Microsystems; Milan, Italy) equipped with a FITC LP filter was used, with an excitation wavelength of 450/490 nm and an exposure time of 30 ms. Paired images of at least five random fields were captured for each sample, encompassing both phase contrast images and FITC filter views.

To identify bacterial cells in the phase contrast images, a custom-written FIJI macro (ImageJ; National Institutes of Health, Bethesda, MD, USA) was employed. Then, we generated a mask and applied it to the FITC channel images. Size filtering and fluorescence intensity measurement were used to eliminate noncellular particulates and background noise. We quantified the number and intensity of each DiBAC-positive cell. The depolarization threshold was established based on the DiBAC average fluorescence intensity from control bacteria and set at the mean value. For each biological replicate and experimental condition, we defined as a final readout the percentage of depolarized cells (% depolarized cells) as the percentage of cells on FITC images surpassing the depolarization threshold relative to phase contrast images. We constructed histogram plots (grouped by experimental group by combining data from different replicates of the same condition) to visualize the bioelectrical profile of each experimental condition. Three independent biological replicates, each with three technical replicates, and a minimum of five images per sample per condition were used.

### 4.6. Statistical Analysis

We compared the bacterial response (i.e., % depolarized cells, OD600, cultivability, and viability) among the different experimental conditions using generalized estimating equations (GEEs). These models utilize the biological replicate as the grouping variable (panel variable) to account for potential data dependencies. For OD600, cultivability, and viability, to compare values among groups, we used multilevel linear regression model (MLiRM). For % depolarized cells, for which the primary objective was to analyze experimental variations in the proportion of depolarized cells among different experimental groups (treatments or times), we used *logit* as link function, thus creating multilevel logistic regression models (MLoRMs).

The obtained results, including statistical analyses, *p*-values, and the number of replicate measurements (N), are stated in the Results Section and in each corresponding figure legend. For detailed statistics, including coefficient values for regression models and confident intervals, see the Supplementary Tables in the Supplementary Materials. Ensuring the utilization of a minimum of three biological replicates, each result is composed of three technical replicates, unless otherwise specified. Data are presented as mean ± standard error of the mean (SEM), and a significance level of 0.05 was applied to all analyses conducted.

To perform statistical analyses and graphical representations, we used STATA 2017 (Stata Statistical Software: Release 15, College Station, TX, USA) and GraphPad Prism v. 8.0.2 (GraphPad Software, Inc., Boston, MA, USA).

## Data Availability

Further information and requests for reagents may be directed to, and will be fulfilled by, the Lead Contact Celia Herrera-Rincon (ceherrer@ucm.es) and María Teresa García-Esteban (mariat16@ucm.es).

## Supplementary Materials

The following supporting information can be downloaded at: https://www.mdpi.com/article/10.3390/ijms25116233/s1.
