# Supplementary material for: From the Microbiome to the Electrome: Implications for the Microbiota–Gut–Brain Axis
Source: Int J Mol Sci. 2024 Jun 5;25(11):6233. doi: 10.3390/ijms25116233 (PMC11172653; doi:10.3390/ijms25116233)
Supplement: Supplementary file 1 [file ijms-25-06233-s001.zip › ijms-2981064-supplementary.pdf]

Supplementary Data

SUPPLEMENTARY FIGURES

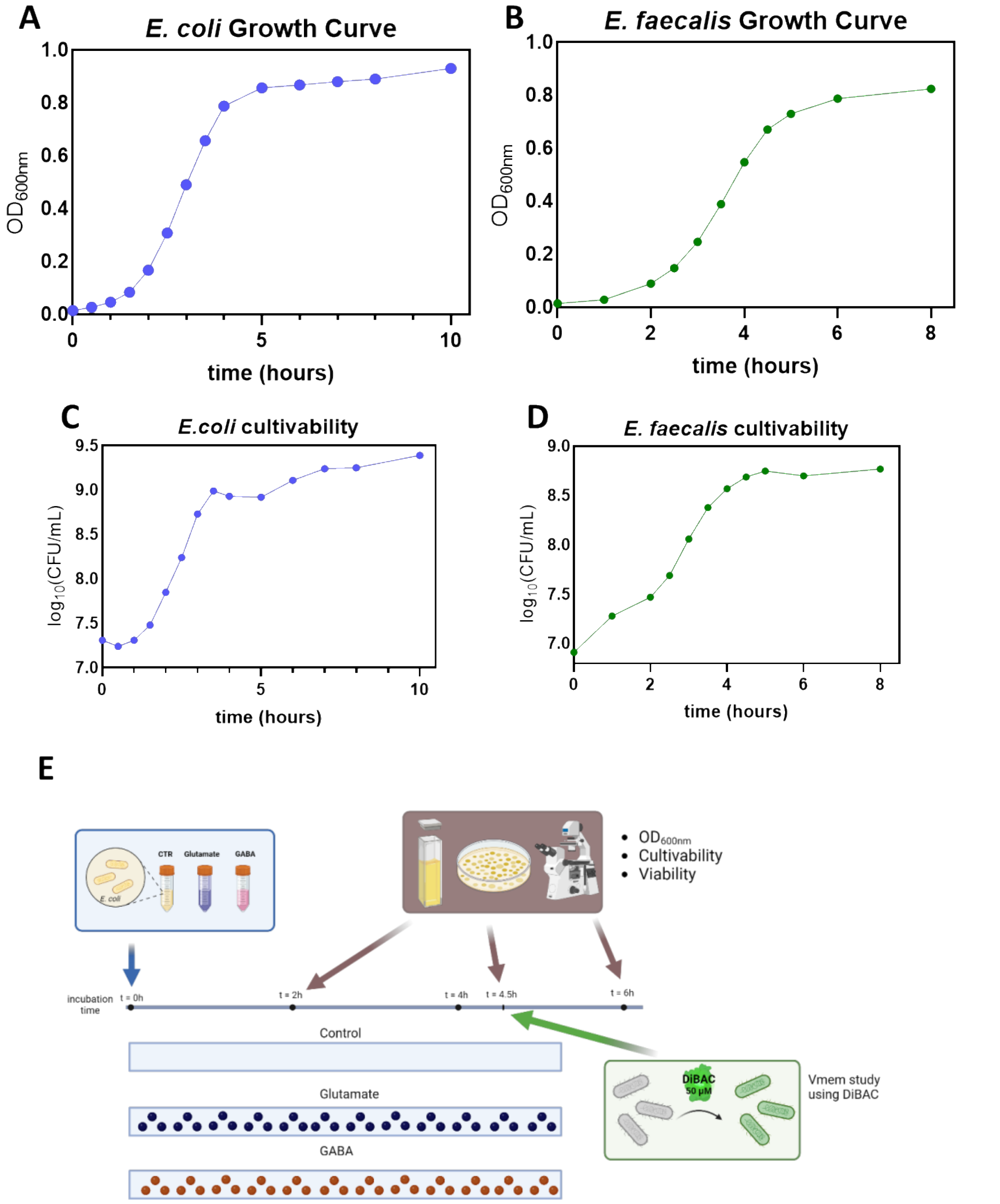

Supplementary Figure S1: (A,B) Growth dynamics of *Escherichia coli (E. coli)* and *Enterococcus faecalis (E. faecalis)*, respectively, (growing in Trypticasein Soy Broth (TSB) medium, aerobic conditions,) in terms of optical density at 600 nm (OD600). **(C,D)** Cultivability of *E. coli* and *E. faecalis*, respectively measured by counting the colony forming units per milliliter (CFU/mL) (Growing in Trypticasein Soy agar (TSA) medium, aerobic conditions). **(E)** Summary timeline of the experiments carried out. At t=0 hours the bacteria were incubated with Glutamate and GABA, plus a control culture

with no treatment. At t=2, 4.5 and 6 hours cultivability was measured; and at t=4.5 hours, bioelectricity and viability were measured.

SUPPLEMENTARY TABLES

Supplementary Table S1. Statistical generalized estimating equations (GEE) results for DiBAC validation assay in *E. coli* and *E. faecalis*

	Condition	KCl 15 mM	KCl 60mM
E. coli	**Control (KCl 0mM)**	<0.001<br>0.3346523<br>[0.253, 0.4163]<br>1.211	<0.001<br>0.9859329<br>[0.9064, 1.0654]<br>2.048
	**KCl 15 mM**		<0.001<br>0.6512806<br>[0.5771, 0.7254]<br>1.692
E. faecalis	**Control (KCl 0mM)**	<0.001<br>0.2689152<br>[0.2157, 0.3221]<br>1.027	<0.001<br>0.4016521<br>[0.3492, 0.4541]<br>1.165
	**KCl 15 mM**		<0.001<br>0.132737<br>[0.0794, 0.1861]<br>1.134

Legend
P value
Coefficient<br>[$CI_{95\%}$ Coefficient
Relative Risk

Outcomes from the GEE analysis regarding the effect of [KCl] on the depolarization ability of *E. coli* and *E. faecalis* cells, with biological replicates serving as the grouping factor. The Relative Risk (RR) is calculated from the minimal concentration value (KCl 0 mM), predicting the alteration upon shifting to the next condition.

Supplementary Table S2: Statistical generalized estimating equations (GEE) results for the study of the bioelectrical analysis in *E. coli* and *E. faecalis* growth dynamics.

	Time	3 hours	5 hours
E. coli	**1 hour**	<0.001<br>-1.6694<br>[-1.8393, -1.4996]<br>0.2746	<0.001<br>-2.2641<br>[-2.4408, -2.0876]<br>0.1591
	**3 hours**		<0.001<br>-0.5947<br>[-0.7242, -0.4653]<br>0.5793
E. faecalis	**1 hour**	<0.001<br>0.6513061<br>[0.3437, 0.9589]<br>1.5246	<0.001<br>1.248706<br>[0.9669, 1.5305]<br>2.0521
	**3 hours**		<0.001<br>0.5973997<br>[0.4016, 0.7932]<br>1.3459

Legend
P value
Coefficient<br>[$CI_{95\%}$ Coefficient
Relative Risk

Outcomes of the GEE analysis on how time impacts the depolarization ability of *E. coli* and *E. faecalis* cells, with biological replicates used as a grouping factor. The Relative Risk (RR) derives from the minimum time value (t=1 h) and predicts the adjustment upon transitioning to the subsequent time point.

Supplementary Table S3: Statistical generalized estimating equations (GEE) results for the study of the effect of neurotransmitters on *E. coli* and *E. faecalis* bioelectricity.

	Treatment	Glutamate	GABA
E. coli	**Control**	<0.001<br>-0.5746<br>[-0.7129, -0.4364]<br>0.6012	<0.001<br>-0.9299<br>[-1.093, -0.7669]<br>0.4327
	**Glutamate**		<0.001<br>-0.3552<br>[-0.5247,-0.1858]<br>0.7198
E. faecalis	**Control**	<0.001<br>-0.3504021<br>[-0.4301, -0.2707]<br>0.8078	<0.001<br>-0.6003356<br>[-0.6822, -0.5185]<br>0.6819
	**Glutamate**		<0.001<br>-0.2499336<br>[-0.3338, -0.1659]<br>0.8441

Legend
P value
Coefficient<br>[$CI_{95\%}$ Coefficient]
Relative Risk

Findings from the GEE analysis on how the timing of neurotransmitter presence affects the depolarization ability of *E. coli* and *E. faecalis* cells, using biological replicates as a categorization factor. The Relative Risk (RR) originates from the control value, assessing the modification upon transitioning to the next treatment.

Neurotransmitters effect on Growth

Supplementary Table S4. Statistical generalized estimating equations (GEE) results for the study of the effect of neurotransmitters on *E. coli* growth (OD600).

Time	Treatment	Glutamate	GABA
**t = 2h**	**Control**	0.164<br>-0.0080<br>[-0.0193, 0.0033]	0.202<br>0.0073<br>[-0.0186, 0.0039]
	**Glutamate**		0.908<br>0.0006<br>[-0.0106, 0.0119]
**t = 4.5h**	**Control**	0.948<br>-0.0003<br>[-0.0103, 0.0096]	0.395<br>0,0043<br>[-0.0056, 0.0143]
	**Glutamate**		0.359<br>0.0046<br>[-0.0053, 0.0146]
**t = 6h**	**Control**	0.697<br>-0.001<br>[-0.006, 0.004]	0.436<br>0.002<br>[-0.003, 0.007]
	**Glutamate**		0.243

			0.003<br>[-0.0020, 0.0080]

Supplementary Table S5. Statistical generalized estimating equations (GEE) results for the study of the effect of neurotransmitters on *E. faecalis* growth (OD600).

Time	Treatment	Glutamate	GABA
t = 2h	Control	0.140<br>-0.0066<br>[-0.0155, 0.0022]	0.712<br>0.0016<br>[-0.0072, 0.0105]
	Glutamate		0.065<br>0.0083<br>[-0.0005, 0.0171]
t = 4.5h	Control	0.215<br>-0.0086<br>[-0.0224, 0.005]	0.703<br>0.0026<br>[-0.011, 0.0164]
	Glutamate		0.105<br>0.0113<br>[-0.0023, 0.0250]
t = 6h	Control	0.270<br>0.0066<br>[-0.0185, 0.0052]	0.440<br>0.0046<br>[-0.0072, 0.0165]
	Glutamate		0.061<br>0.0113<br>[-0.0005, 0.02317]

Legend
P value
Coefficient<br>[$CI_{95\%}$ Coefficient]

Results for the GEE analysis of the influence of the Neurotransmitters on the growth of *E. coli* (S4) and *E. faecalis* (S5) cells, considering the biological replicate as a grouping variable. No significant differences were found

Neurotransmitters effect on cultivability supplementary tables

Supplementary Table S6. Statistical generalized estimating equations (GEE) results for the study of the effect of neurotransmitters on *E. coli* cultivability along its growth curve

Time	Treatment	Glutamate	GABA
t = 2h	Control	0.851<br>-0.0003<br>[-0.0038, 0.0032]	0.708<br>0.0006<br>[-0.0042, 0.0028]
	Glutamate		0.851<br>-0.0003<br>[-0.0038, 0.0031]
t = 4.5h	Control	0.516<br>0.001<br>[-0.002, 0.004]	0.829<br>-3.00e-15<br>[-0.0027, 0.0034]
	Glutamate		0.665<br>0.0016665<br>[-0.0036, 0.0023]
t = 6h	Control	0.275<br>0.0016<br>[-0.0013, 0.0047]	1<br>-0.0003<br>[-0.003, 0.003]
	Glutamate		0.275<br>0.0016<br>[-0.0046, 0.0013]

Legend
P value
Coefficient<br>[$CI_{95\%}$ Coefficient]

Supplementary Table S7: Statistical generalized estimating equations (GEE) results for the study of the effect of neurotransmitters on *E. faecalis* cultivability along its growth curve

Time	Treatment	Glutamate	GABA
t = 2h	Control	0.853<br>-0.001<br>[-0.0116, 0.0096]	0.853<br>0.001<br>[-0.0096, 0.0116]
	Glutamate		0.710<br>0.002<br>[-0.0085, 0.0125]
t = 4.5h	Control	0.549<br>0.0016<br>[-0.0071, 0.0038]	0.99<br>-9.54e-08<br>[-0.0055, 0.0055]
	Glutamate		0.549<br>-0.0006667<br>[-0.0037, 0.0071]
t = 6h	Control	0.942<br>-0.0333<br>[-0.9353, 0.8686]	0.346<br>0.4333<br>[-0.4686, 1.3353]
	Glutamate		0.311<br>0.4666<br>[-0.4352, 1.3686]

Results for the GEE analysis of the influence of the Neurotransmitters on the cultivability of *E. coli* (S6) and *E. faecalis* (S7) cells, considering the biological replicate as a grouping variable. No significant differences were found

Neurotransmitters effect on viability supplementary tables

Supplementary Table S8: Statistical generalized estimating equations (GEE) results for the study of the effect of neurotransmitters on E. coli viability.

Legend
P value
Coefficient [$CI_{95\%}$ Coefficient]

	Treatment	Glutamate	GABA
**E. coli**	**Control**	0.662<br>0.1165<br>[-0.4068, 0.64]	0.059<br>0.5139<br>[-0.0188, 1.0467]
	**Glutamate**		0.144<br>0.3973<br>[0.1353, 0.9301]
**E. faecalis**	**Control**	0.110<br>-0.6873<br>[-1.5296, 0.1549]	0.791<br>0.1100<br>[-0.7027, 0.9228]
	**Glutamate**		0.062<br>0.7974<br>[0.0414, 1.6363]

Results for the GEE analysis of the influence of the Neurotransmitters on the viability of *E. coli* and *E. faecalis* cells, considering the biological replicate as a grouping variable. No significant differences were found

SPECIFIC GROWTH RATE or r_t

Legend:

rt is the specific growth rate, a parameter that we use as an indicator of the physiological state of the bacterial cells, and it is defined by the following equation:

where r = growth rate

$$r_t = r\left(1 - \frac{OD_t}{k}\right)$$

OD_t = Optical Density values at each measured time

k = estimated carrying capacity of the culture

For *E. coli* r (1.41649) and *k* (0.904673), and for *E. faecalis* r(1.08761) and (0.982551) are calculated by fitting OD data to a Verhulst growth curve, performed by a non-linear regression analysis on the following function:

$$y(t) = \frac{y_0 k \exp{(rt)}}{k + y_0(\exp(rt) - 1)}$$

where y_0=0.010 (estimated from OD600 data)

Nernst EQUATION FOR EQUILIBRIUM POTENTIAL (VEq; Eq.1)

$$V_{Eq.} = \frac{RT}{zF} \ln\left(\frac{[K^+]_{out}}{[K^+]_{in}}\right)$$

where $V_{Eq.}$ is the equilibrium potential;
R is the universal gas constant and is equal to 8.314 $J.K^{-1}.mol^{-1}$ (Joules per Kelvin per mole);
T is the temperature in Kelvin (K = °C + 273.15);
Z is the valence of K^+ (+1; unitless);
F is the Faraday's constant and is equal to 96,485 $C.mol^{-1}$ (Coulombs per mole);
($[K^+]$)t is the concentration of K^+ in the extracellular medium (mM);
($[K^+]$)is the concentration of K^+ in the intracellular medium (mM).
